# Novel therapeutic target against Alzheimer

**DOI:** 10.18632/oncotarget.18210

**Published:** 2017-05-25

**Authors:** Andrea C. LeBlanc

**Affiliations:** Bloomfield Center for Research in Aging, Lady Davis Institute for Medical Research, Sir Mortimer B Davis Jewish General Hospital, Montréal, QC, Canada

**Keywords:** Alzheimer disease, caspase-1, caspase-6, caspase inhibitors, Nlrp1 inflammasome

Novel therapeutic targets against which we can develop medications are urgently needed in the field of Alzheimer Disease (AD) research. While immense progress has been made in the description and diagnosis of AD, no effective medication to prevent or treat AD exists. Yet, 47 million individuals suffer from AD worldwide. The cost to care for AD and other dementias is $259 billion dollars/year in the USA alone.

The current anti-cholinesterase and NMDA receptor antagonist treatments offered to patients have a modest therapeutic effect. In the last 30 years, research to improve treatments has mainly focused on eliminating amyloid, a short peptide that accumulates in AD brains and forms the extracellular amyloid plaques, a hallmark pathology of AD. Unfortunately, despite a multitude of approaches, clinical trials have failed to show efficacy with anti-amyloid treatments. An emerging current indicates more efforts to be directed to target Tau, a conformationally modified and hyperphosphorylated intraneuronal protein that forms the neurofibrillary tangles, the other quintessential neuropathological lesion of AD brains.

While time will tell if these approaches will eventually yield an effective drug, it may be prudent to pursue additional therapeutic targets to increase our chances of developing efficient treatments against AD. Focusing on the identification of tractable earlier molecular mechanisms of neurodegeneration in human age-dependent cognitive impairment and AD, my laboratory has identified a potential novel therapeutic target in Caspase-6 (Casp6).

Casp6, a cysteinal protease belonging to the family of caspase proteins involved in inflammation and programmed cell death, is associated with AD pathology and memory impairment [1]. Active Casp6 and Tau cleaved by Casp6 are abundantly present in the neuritic plaques, neurofibrillary tangles and neuropil threads in the brains of mild, moderate, severe and very severe AD cases. No active Casp6 has been detected in younger brains (≤ 45 yrs). Yet, active Casp6 has been detected in brains from non-cognitively impaired aged individuals. In these cases, active Casp6 is limited mostly to the entorhinal cortex, one of the first areas of the brain affected by neurofibrillary tangles in AD. Casp6 levels correlate with lower episodic and semantic memory performance, two types of memory also affected early in AD [1]. Expression of a self-activated form of Casp6 in mouse hippocampus causes age-dependent spatial and episodic memory impairment [2]. Therefore, Casp6 represents a pre-symptomatic target of age-dependent cognitive decline and AD.

**Figure 1:**
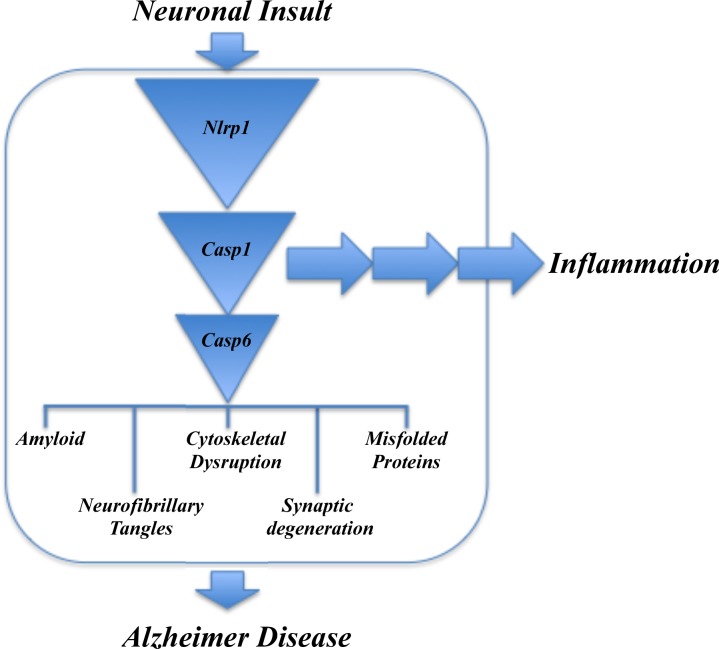
Nlrp1-Casp1-Casp6 neurodegenerative pathway involved in age-dependent cognitive impairment and Alzheimer Disease

At a molecular level, Casp6 activity induces parallel pathways involved in AD neurodegeneration and pathology (Figure 1) [1]. Stress treatments of human CNS primary neurons induce Casp6-mediated increased production of amyloid beta peptide. Casp6 cleaves Tau and alpha-tubulin microtubule-associated proteins, Drebrin, Spinophilin, alpha-Actinin-1 and -4 actin-regulating synaptic proteins, and impairs proteasomal degradation of misfolded proteins by cleaving valosin-containing protein. In human neurons, active Casp6 causes neuritic degeneration [3, 4]. The role of Casp6 in axonal degeneration has been elegantly shown in mouse neuron cultures and in the brain of several mouse models [Nikolaev A, et al. Nature. 2009; 457:981-989; Uribe V, et al. Hum Mol Genet. 2012; 21:1954-1967; Cusack CL, et al. Nat Commun. 2013; 4:1876]. Therefore, inhibiting Casp6 activity may stem neuronal degeneration.

In human neurons, Casp6 is activated through the inflammatory Nlrp1-Casp1 pathway and causes neuritic degeneration [3, 4]. Nlrp1 is increased 25-30 fold in AD neurons and is co-localized with active Casp6, indicating that this Nlrp1-Casp1-Casp6 pathway is active in AD neurons. Most importantly, the Nlrp1-Casp1-Casp6 pathway links a neuron specific inflammatory pathway via Nlrp1 with Casp6-mediated neuronal degeneration. This pathway would not be prevented by current non-steroidal anti-inflammatory treatments, which have not yet shown significant effects in AD clinical trials. Targeting Nlrp1 might be a better anti-inflammatory approach than these non-steroidal anti-inflammatory treatments, but much more basic information is required on Nlrp1 structure and function to assess the feasibility of Nlrp1 as a novel therapeutic target against AD.

Casp6 is likely a good drug target. Adult human brains express the lowest levels of Casp6 zymogen protein compared to other human tissues, suggesting that Casp6 may not normally play an important role in the brain. Casp6 null mice do not have obvious deficits although further probing indicates a role for Casp6 in B cell activation and differentiation into plasma cells [5]. Casp6 also sensitizes mouse sensory nerves to pain via microglial activation [6]. To date, none of the functions attributed to Casp6 indicate potential detrimental effects by Casp6 inhibitors.

Our latest paper shows that Casp6-mediated memory impairment can be reversed in old mice [7]. A vinyl sulfone non-toxic and blood brain barrier permeable small peptide caspase inhibitor reverses Casp6-mediated age-dependent memory impairment in mice. This finding supports the potential of Casp6 inhibitors to restore memory impairment in aged individuals and highlights the importance of developing selective and specific Casp6 inhibitors. Inhibitors targeting the active site of Casp6 may not offer enough selectivity because of the structural similarity amongst the active sites of all caspases. Allosteric regulation may be required to selectively inhibit Casp6 [8], to avoid altering the activity of the other caspases, which are very important in regulating tissue homeostasis.

In conclusion, finding a selective non-toxic and blood brain barrier permeable Casp6 inhibitor is essential to validate Casp6 as an early therapeutic target of neurodegeneration in mouse AD models, and to assess if inhibiting Casp6 can prevent the progressive cognitive impairment and the ensuing dementia in AD.
